# Defining Posttraumatic Sepsis for Population-Level Research

**DOI:** 10.1001/jamanetworkopen.2022.51445

**Published:** 2023-01-18

**Authors:** Katherine Stern, Qian Qiu, Michael Weykamp, Grant O’Keefe, Scott C. Brakenridge

**Affiliations:** 1 Division of Trauma, Burn, and Critical Care, Department of Surgery, University of Washington, Seattle; 2 University of Washington School of Public Health, Seattle; 3 University of San Francisco East Bay General Surgery Residency Program, Oakland, California; 4 Harborview Injury Prevention Center, University of Washington, Seattle

## Abstract

**Importance:**

Multiple classification methods are used to identify sepsis from existing data. In the trauma population, it is unknown how administrative methods compare with clinical criteria for sepsis classification.

**Objectives:**

To characterize the agreement between 3 approaches to sepsis classification among critically ill patients with trauma and compare the sepsis-associated risk of adverse outcomes when each method was used to define sepsis.

**Design, Setting, and Participants:**

This retrospective cohort study used data collected between January 1, 2012, and December 31, 2020, from patients aged 16 years or older with traumatic injury, admitted to the intensive care unit of a single-institution level 1 trauma center and requiring invasive mechanical ventilation for at least 3 days. Statistical analysis was conducted from August 1, 2021, to March 31, 2022.

**Exposure:**

Hospital-acquired sepsis, as classified by 3 methods: a novel automated clinical method based on data from the electronic health record, the National Trauma Data Bank (NTDB), and explicit and implicit medical billing codes.

**Main Outcomes and Measures:**

The primary outcomes were chronic critical illness and in-hospital mortality. Secondary outcomes included number of days in an intensive care unit, number of days receiving mechanical ventilation, discharge to a skilled nursing or long-term care facility, and discharge to home without assistance.

**Results:**

Of 3194 patients meeting inclusion criteria, the median age was 49 years (IQR, 31-64 years), 2380 (74%) were male, and 2826 (88%) sustained severe blunt injury (median Injury Severity Score, 29 [IQR, 21-38]). Sepsis was identified in 747 patients (23%) meeting automated clinical criteria, 118 (4%) meeting NTDB criteria, and 529 (17%) using medical billing codes. The Light κ value for 3-way agreement was 0.16 (95% CI, 0.14-0.19). The adjusted relative risk of chronic critical illness was 9.9 (95% CI, 8.0-12.3) for sepsis identified by automated clinical criteria, 5.0 (95% CI, 3.4-7.3) for sepsis identified by the NTDB, and 4.5 (95% CI, 3.6-5.6) for sepsis identified using medical billing codes. The adjusted relative risk for in-hospital mortality was 1.3 (95% CI, 1.0-1.6) for sepsis identified by automated clinical criteria, 2.7 (95% CI, 1.7-4.3) for sepsis identified by the NTDB, and 1.0 (95% CI, 0.7-1.2) for sepsis identified using medical billing codes.

**Conclusions and Relevance:**

In this cohort study of critically ill patients with trauma, administrative methods misclassified sepsis and underestimated the incidence and severity of sepsis compared with an automated clinical method using data from the electronic health record. This study suggests that an automated approach to sepsis classification consistent with Third International Consensus Definitions for Sepsis and Septic Shock (Sepsis-3) clinical criteria is feasible and may improve existing approaches to health services and population-based research in this population.

## Key Points

**Question:**

Do existing administrative data methods commonly used for quality and research purposes adequately capture sepsis events in critically ill patients with traumatic injury?

**Findings:**

In this cohort study that included 3194 adults, using automated clinical database (Third International Consensus Definitions for Sepsis and Septic Shock [Sepsis-3]) criteria identified significantly more cases of sepsis, with worse morbidity outcomes, compared with using the National Trauma Data Bank and administrative coding methods.

**Meaning:**

The results of this study suggest that administrative classification methods misclassify and underestimate sepsis events among severely injured patients with trauma, suggesting that new analytic approaches are needed for this population.

## Teaser

This cohort study characterizes the agreement between 3 approaches to sepsis classification among critically ill patients with trauma and compares the sepsis-associated risk of adverse outcomes when each method is used to define sepsis.

## Introduction

Despite advances in surveillance, protocolized resuscitation, and critical care organ support, hospital-acquired sepsis remains a prevalent complication and contributor to morbidity and mortality after severe traumatic injury.^1,2,3,4^ Thus, the prevention, early detection, and effective treatment of sepsis after injury are of great research interest. The Third International Consensus Definitions for Sepsis and Septic Shock (Sepsis-3) consensus guidelines provide a conceptual framework for the clinical diagnosis of sepsis by the physician at the bedside^5,6^; however, the accurate classification of sepsis from administrative and/or clinical data for quality analysis and clinical outcomes research remains a challenge, and multiple methods are used in practice.^7,8,9^

In the trauma population, the American College of Surgeons National Trauma Data Bank (NTDB) definition for severe sepsis has been the reference standard for population-level trauma studies and continues to be used in clinical outcomes research.^10,11^ The epidemiology of posttraumatic sepsis has also been reported using explicit and implicit criteria from medical billing codes.^3,12^ The NTDB and medical billing code databases are convenient resources for population-level studies. However, coding practices and sepsis definitions change over time; as such, methods using the NTDB and medical billing code databases can misclassify sepsis according to contemporary (ie, Sepsis-3) definitions.^13,14,15,16^ Sepsis adjudication by clinician review of the medical record is viewed as the preferred method for sepsis classification.^1,15,17,18^ However, even when rubrics and a consensus process between reviewers are used, adjudication introduces subjectivity, and results may be difficult to reproduce. Medical record review is also time-consuming and may not be feasible when working with large data sets.^19^

To improve the reproducibility and efficiency of sepsis classification, there is growing interest in automating the process using clinical data from the electronic medical record (EMR).^9,14,20^ With an automated classification strategy, we can apply the same diagnostic criteria to data sets from different centers and over different time periods.^1,6,14,17,21^ In addition, criteria can be modified to account for coexisting acute and/or chronic comorbidities that may overlap with and obscure the clinical criteria for Sepsis-3.^20^ This adaptability may be useful for classifying sepsis among patients with injury-associated systemic inflammation and organ dysfunction.^17,22^ To our knowledge, an automated approach to sepsis classification has not been evaluated for the trauma population.

We applied an automated method for sepsis classification adapted from the Centers for Disease Control and Prevention’s adult sepsis surveillance criteria^23^ and the original Sepsis-3 guidelines^6^ to a cohort of critically ill patients with trauma. We then characterized the reliability of sepsis classifications from an automated clinical method, the NTDB, and the medical billing code method described by Angus and colleagues,^12,19^ and we evaluated the implications of method choice on the epidemiology of sepsis after injury.

## Methods

### Setting and Design

We conducted a retrospective cohort study of individuals admitted to Harborview Medical Center (Seattle, Washington) between January 1, 2012, and December 31, 2020. Harborview Medical Center serves as the sole level 1 trauma center for 4 states (Alaska, Washington, Montana, and Idaho) and receives more than 6000 trauma admissions per year. This study was classified as minimal risk and approved by the University of Washington institutional review board with a waiver of informed consent. The study design and reporting are consistent with the Strengthening the Reporting of Observational Studies in Epidemiology (STROBE) reporting guideline.

### Study Population

We included patients with injuries from blunt force or penetrating trauma who were aged 16 years or older, admitted to the intensive care unit (ICU), and required invasive mechanical ventilation for at least 3 days. Patient demographic characteristics, including race and ethnicity, were recorded by study coordinators after enrollment, as per National Institutes of Health definitions and requirements. We excluded individuals whose first inpatient hospital unit was a location other than the ICU or an emergency procedural unit (eg, operating room or angiography) (eFigure 1 in Supplement 1).

### Sepsis Definitions

We defined sepsis consistent with the Sepsis-3 consensus guidelines as a clinically suspected infection associated with acute worsening of organ dysfunction.^5,24^ To identify posttraumatic sepsis retrospectively (automated clinical method), we used the Centers for Disease Control and Prevention’s adult sepsis surveillance criteria^14^ with a priori modifications using readily obtainable EMR data to address classification challenges specific to the trauma population. We required that all of the following conditions be present: (1) an order for a new intravenous or qualifying oral antibiotic, not administered within the previous 48 hours and excluding antibiotics used for surgical prophylaxis, at our institution; (2) a body tissue culture was ordered within 48 hours of antibiotic initiation; (3) a qualifying antibiotic was administered for at least 4 consecutive days or until death or discharge; and (4) a 2-point increase in the maximum daily Sequential Organ Failure Assessment (SOFA) score occurred within 3 days before and 3 days after the qualifying tissue culture (eTable 1 and eMethods in Supplement 1).

We ascertained sepsis using 2 additional classification methods: the NTDB and the method described by Angus and colleagues.^12^ The NTDB classification of severe sepsis is performed by a nonclinician administrator at each contributing NTDB institution and includes manual record review for documentation of sepsis as defined by the 2017 NTDB data dictionary (ie, consistent with the Sepsis-2 definitions of *severe sepsis* and *septic shock*).^10^ The method described by Angus et al^12^ (administrative method) includes explicit and implicit criteria for sepsis based on *International Classification of Disease* codes (sepsis classification methods are described in detail in eTable 1 and the eMethods in Supplement 1).

### Outcomes

The primary outcomes were chronic critical illness, defined as being alive in the ICU with a SOFA score of 3 or higher on hospital days 13, 14, or 15,^25^ and in-hospital mortality, defined as death during the same admission. Secondary outcomes included number of days in an ICU, number of days receiving mechanical ventilation, discharge to a skilled nursing or long-term care facility, and discharge to home without assistance.

### Covariates

We constructed a directed acyclic graph^26^ to guide the selection of covariates associated with posttraumatic sepsis and the primary outcomes. These covariates included patient age in years; preexisting comorbidities^27^; Injury Severity Score; the presence of severe head injury (maximum head Abbreviated Injury Severity Score ≥3); multiple traumatic injuries^28^; the modified SOFA score from the first day of hospitalization; shock on admission, defined as a base deficit of 6 mEq/L of bicarbonate (to convert to millimoles per liter, multiply by 1.0) during the first 24 hours or an admission systolic blood pressure less than 90 mm Hg^29^; and units of red blood cells transfused during the first 24 hours. For descriptive analyses, we also collected information on patient biological sex, injury mechanism (blunt or penetrating), body regions with severe injuries (Abbreviated Injury Severity Score ≥3), the simplified Acute Physiology and Chronic Health Evaluation (APACHE II physiology components) score, and emergency laparotomy. All data were obtained from our center’s EMR and trauma registry.

### Statistical Analysis

Statistical analysis was conducted from August 1, 2021, to March 31, 2022. We characterized the overall and pairwise agreement between the 3 classification methods using the Light κ value. We computed the 95% CI for the estimated 3-way κ value using a bootstrap method with 1000 replicates.^30^ We prespecified a κ value of less than 0.75 as good agreement, a κ value of 0.5 to 0.75 as moderate, and a κ value of less than 0.5 as poor.^31^

For each sepsis classification method, we compared the characteristics and outcomes of patients who were classified with sepsis with patients who were not classified with sepsis using the Wilcoxon rank sum test or the Pearson χ^2^ test. We corrected *P* values for multiple testing within each method-based comparison using Bonferroni adjustments.

We used univariate and multivariable log-binomial models to estimate the unadjusted and adjusted relative risks (RRs) and 95% CIs of chronic critical illness and of in-hospital mortality associated with sepsis when classified by each method. To compute the RR and 95% CI, we used robust SE estimates based on our assumption that the variances between groups are not truly equal. We considered sepsis identified by the automated clinical method on or before hospital day 10 separately to distinguish sepsis events that occurred during the acute phase of recovery from those that coincided with or occurred during a protracted ICU stay.

We used Cox proportional hazards regression models to estimate the unadjusted and adjusted hazard ratios of resolving organ dysfunction (SOFA score <3 sustained for ≥3 days or until discharge alive) and in-hospital mortality at any point after sepsis diagnosis, comparing patients with sepsis with patients at risk. For time-to-event analyses, we created separate risk sets wherein individuals contributed time at risk to the no sepsis group prior to sepsis onset (see the eMethods in Supplement 1 for additional details).

All hypothesis tests were 2-sided and performed using an α level of .05. Data processing was performed in Stata, version 16.1 (StataCorp LLC); statistical analyses and visualizations were performed in R, version 4.1.0 (R Group for Statistical Computing), with the eulerr, irr, psych, boot, and rigr packages.

## Results

Of 3194 individuals who met inclusion criteria, the median age was 49 years (IQR, 31-64 years), 2380 (74%) were male, and 2826 (88%) sustained blunt force trauma (median Injury Severity Score, 29 [IQR, 21-38]) (Table 1). Most patients sustained injuries to multiple body regions (1868 [58%]), 1551 (49%) had severe traumatic brain injury, and 1266 (40%) received red blood cell transfusions during the first 24 hours. Additional patient characteristics are reported in Table 1.

**Table 1.** Characteristics and Clinical Outcomes of Critically Ill Injured Patients Admitted to Harborview Medical Center Intensive Care Unit (2012-2020)

Characteristic	No. (%) (N = 3194)^a^
Age, median (IQR), y	49 (31-64)
Sex	
Female	814 (26)
Male	2380 (74)
Race	
American Indian or Alaska Native	117 (4)
Asian	184 (6)
Black	247 (8)
White	2403 (75)
Other race^b^	33 (1)
Not documented	210 (7)
Ethnicity	
Hispanic	277 (9)
Non-Hispanic	2917 (91)
Charlson Comorbidity Index	
0	1549 (49)
1-2	944 (30)
3-4	500 (16)
≥5	201 (6)
Mechanism of injury	
Blunt	2826 (88)
Penetrating	368 (12)
Injury Severity Score, median (IQR)	29 (21-38)
Body regions with an AIS ≥3^c^	
Head	1551 (49)
Neck	338 (11)
Chest	1578 (49)
Abdomen	700 (22)
Spine	656 (21)
Lower extremity	975 (31)
Multiple trauma^d^	1868 (58)
Base deficit, median (IQR), mEq/L of bicarbonate^e^	5 (3-8)
Unknown	14
Initial ED SBP <90 mm Hg	408 (13)
RBC transfusion within first 24 h	
None	1928 (60)
1-4 Units	752 (24)
5-9 Units	303 (10)
≥10 Units	211 (7)
APACHE II score, median (IQR)^f^	27 (22-32)
Emergency laparotomy	584 (18)
Outcomes	
ICU days, median (IQR), No.	10 (6-17)
Mechanical ventilation days, median (IQR), No.	6 (4-12)
Chronic critical illness	520 (16)
Died in hospital	637 (20)
Discharged to SNF or LTCF	780 (24)
Discharged to home without assistance	609 (19)

Abbreviations: AIS, Abbreviated Injury Severity Score; APACHE II, Acute Physiology and Chronic Health Evaluation; ED, emergency department; ICU, intensive care unit; LTCF, long-term care facility; RBC, red blood cell; SBP, systolic blood pressure; SNF, skilled nursing facility.

^a^ Missing values greater than 5% are reported.

^b^ Includes Native Hawaiian or Other Pacific Islander.

^c^ Body regions with frequency less than 10% are not shown and included face, upper extremity, and external injuries.

^d^ Defined as at least 2 body regions with serious injury (AIS ≥3) and at least 1 of the following physiological abnormalities during the first 2 calendar days of admission: Glasgow Coma Scale score of 8 or lower, base excess of 6 mEq/L or less of bicarbonate (to convert to millimoles per liter, multiply by 1.0), SBP less than 90 mm Hg, international normalized ratio of 1.4 or more, and aged 70 years or older.

^e^ Highest value documented during the first 48 hours of admission.

^f^ Includes physiology components and age; omits comorbidities.

### Agreement Between Classification Methods

Sepsis was identified in 747 patients (23%) according to the automated clinical method, 118 patients (4%) according to the NTDB, and 529 patients (17%) according to the administrative method. The overall agreement between methods was poor (Light κ value, 0.16 [95% CI, 0.14-0.19]). Pairwise agreement was also poor (automated clinical method and NTDB: Light κ value, 0.13 [95% CI, 0.10-0.16]; automated clinical method and administrative method: Light κ value, 0.28 [95% CI, 0.24-0.32]; and NTDB and administrative method: Light κ value, 0.08 [95% CI, 0.05-0.12]). Annual sepsis prevalence and between-method agreement are shown in the Figure.

**Figure.**
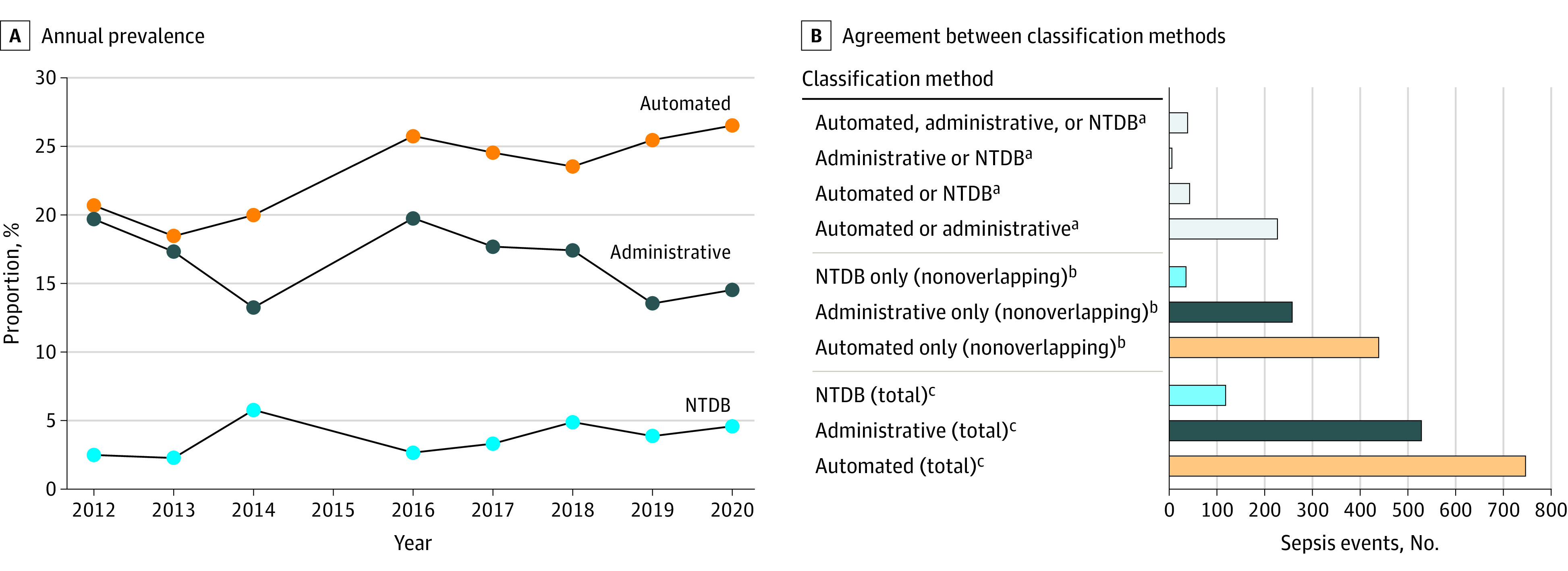
Prevalence of Sepsis According to 3 Classification Methods and Agreement Between the Methods A, Annual prevalence of posttraumatic sepsis according to 3 classification methods: an automated method using clinical data from the electronic medical record (Automated), the National Trauma Data Bank (NTDB), and a medical billing code method described by Angus and colleagues^12^ (Administrative). B, Agreement between the 3 sepsis classification methods. Annual prevalence is not reported for 2015 because study data did not include May through December of 2015. ^a^Overlapping sepsis classifications. ^b^Sepsis cases classified only within each classification method (nonoverlapping cases). ^c^Total number of sepsis cases meeting each classification’s criteria.

### Epidemiology of Posttraumatic Sepsis According to 3 Classification Methods

According to the automated clinical method, 88% of sepsis events (654 of 747) developed during the first 14 days of hospitalization (Table 2). The most common types of infection were respiratory (342 of 747 [46%]), followed by culture-negative infections (248 of 747 [33%]), bloodstream infections (89 of 747 [12%]), and surgical site or traumatic wounds (64 of 747 [9%]). Injury-associated organ dysfunction was prevalent in the cohort, with a median SOFA score of 3 (IQR, 2-6) at the presepsis baseline (ie, the day prior to the organ dysfunction window). Recurrent sepsis (ie, >1 Sepsis-3 event) was common (257 of 747 [34%]). The timing of antibiotic administration and organ dysfunction relative to the first qualifying culture are presented in Table 2. Sepsis event details were not available using the NTDB or the administrative methods.

**Table 2.** Characteristics of the First Sepsis-3 Event Identified by an Automated Clinical Method for Sepsis Classification

Characteristic	No. (%) (N = 747)
Timing of first sepsis event	
Days 3-7	372 (50)
Days 8-14	282 (38)
Days >14	93 (12)
Infections^a^	
Respiratory	342 (46)
Culture negative	248 (33)
Blood	89 (12)
Surgical or injury site	64 (9)
Genitourinary	42 (6)
Cerebrospinal fluid	3 (0.4)
Timing of new intravenous antibiotic relative to culture	
1-2 Days before	37 (5)
Same day	500 (67)
1 Day after	169 (23)
2 Days after	41 (6)
Timing of acute SOFA score change	
Early (2-3 d before culture)	194 (26)
Middle (2 d before-1 d after culture)	462 (62)
Late (2-3 d after culture)	91 (12)
SOFA score, median (IQR)	
Day prior to organ dysfunction window	3 (2-6)
First day of organ dysfunction window	3 (1-5)
Multiple sepsis events^b^	257 (34)

Abbreviations: SOFA, Sequential Organ Failure Assessment; Sepsis-3, Third International Consensus Definitions for Sepsis and Septic Shock.

^a^ Percentages of infections total more than 100% because any culture that met site-specific criteria for infection is listed as a potential source. Culture negative indicates that no cultures during the sepsis event met criteria for infection.

^b^ Met all criteria on more than 1 nonconsecutive day.

Individuals classified with sepsis according to any of the 3 methods had similar prehospital characteristics, higher indices of injury severity and acute physiological derangement, and worse morbidity outcomes compared with their counterparts without sepsis (Table 3). However, injury pattern and severity were similar across sepsis classification methods. Maximum daily SOFA scores were, on average, higher among patients classified with sepsis by all 3 methods, followed by patients classified by the NTDB and the automated clinical method, patients classified by the administrative method, and patients meeting no sepsis criteria, respectively (eFigure 2 in Supplement 1).

**Table 3.** Comparison of Patient Characteristics and Clinical Outcomes of Injured, Critically Ill Adults With or Without Posttraumatic Sepsis According to 3 Classification Methods

Characteristic	Automated clinical method^a^			NTDB^a^			Administrative method^a^		
	No sepsis (n = 2447)	Sepsis (n = 747)	*P* value^b^	No sepsis (n = 3076)	Sepsis (n = 118)	*P* value^b^	No sepsis (n = 2665)	Sepsis (n = 529)	*P* value^b^
Prehospital									
Age, median (IQR), y	49 (31-64)	49 (32-63)	>.99	49 (31-64)	55 (37-68)	.50	49 (31-64)	52 (32-64)	>.99
Sex, No. (%)									
Female	647 (26)	167 (22)	.60	781 (25)	33 (28)	>.99	688 (26)	126 (24)	>.99
Male	1800 (74)	580 (78)		2295 (75)	85 (72)		1977 (74)	403 (76)	
Race, No. (%)									
American Indian or Alaska Native	87 (4)	30 (4)	>.99	113 (4)	3 (3)	>.99	101 (4)	16 (3)	>.99
Asian	145 (6)	39 (5)		173 (6)	11 (9)		148 (6)	36 (7)	
Black	197 (8)	50 (7)		242 (8)	5 (4)		212 (8)	35 (7)	
White	1831 (75)	572 (77)		2321 (75)	82 (69)		2002 (75)	401 (76)	
Other race	24 (1)	9 (1)		30 (1)	3 (3)		27 (1)	6 (1)	
Not documented	163 (7)	47 (6)		197 (6)	13 (11)		175 (7)	35 (7)	
Ethnicity, No. (%)									
Hispanic	213 (9)	64 (9)	>.99	264 (9)	13 (11)	>.99	239 (9)	38 (7)	>.99
Non-Hispanic	2234 (91)	683 (91)		2812 (91)	105 (89)		2426 (91)	491 (93)	
Charlson Comorbidity Index ≥3	551 (23)	150 (20)	>.99	666 (22)	35 (30)	>.99	580 (22)	121 (23)	>.99
Injury									
Blunt force mechanism, No. (%)	2143 (88)	683 (91)	.09	2729 (89)	97 (82)	.70	2347 (88)	479 (91)	>.99
Injury Severity Score, median (IQR)	27 (21-38)	30 (25-43)	<.001	29 (21-38)	29 (25-38)	>.99	27 (21-38)	30 (25-43)	<.001
Body regions with an AIS ≥3, No. (%)^c^									
Head	1182 (48)	369 (49)	>.99	1514 (49)	37 (31)	.003	1332 (50)	219 (41)	.007
Chest	1150 (47)	428 (57)	<.001	1515 (49)	63 (53)	>.99	1269 (48)	309 (58)	<.001
Abdomen	490 (20)	210 (28)	<.001	656 (21)	44 (37)	<.001	534 (20)	166 (31)	<.001
Spine	494 (20)	162 (22)	>.99	635 (21)	21 (18)	>.99	528 (20)	128 (24)	.50
Lower extremity	733 (30)	242 (32)	>.99	925 (30)	50 (42)	.10	770 (29)	205 (39)	<.001
Multiple trauma^d^	1386 (57)	482 (65)	.003	1789 (58)	79 (67)	>.99	1518 (57)	350 (66)	.002
Physiology and interventions									
Base deficit, median (IQR), mEq/L of bicarbonate^e^	5 (3-8)	6 (3-9)	.02	5 (3-8)	7 (4-10)	.02	5 (3-8)	6 (4-10)	<.001
Missing, No.	14	14	NA	14	14	NA	15	10	NA
Initial ED SBP <90 mm Hg, No. (%)	302 (13)	106 (14)	>.99	385 (13)	23 (20)	.50	309 (12)	99 (19)	<.001
Admission SOFA score, median (IQR)^f^	3 (1-5)	4 (2-6)	<.001	3 (1-5)	3 (2-6)	>.99	3 (1-5)	4 (2-6)	<.001
RBC transfusion within first 24 h, No. (%)	924 (38)	342 (46)	.002	1198 (39)	68 (58)	.001	1012 (38)	254 (48)	<.001
RBC units transfused within first 24 h, median (IQR)	3 (1-6)	3 (1-8)	.004	3 (1-6)	4 (2-12)	.02	3 (1-6)	4 (2-8)	<.001
Emergency laparotomy, No. (%)	399 (16)	185 (25)	<.001	538 (17)	46 (39)	<.001	441 (17)	143 (27)	<.001
Outcomes									
ICU days, median (IQR)	8 (6-13)	19 (13-29)	<.001	10 (6-17)	18 (13-31)	<.001	9 (6-15)	19 (11-30)	<.001
Mechanical ventilation days, median (IQR)	5 (3-8)	14 (9-21)	<.001	6 (4-11)	13 (7-21)	<.001	5 (4-10)	13 (7-22)	<.001
Chronic critical illness, No. (%)^g^	186 (8)	334 (45)	<.001	460 (15)	60 (51)	<.001	311 (12)	209 (40)	<.001
Died in hospital, No. (%)	463 (19)	174 (23)	.20	595 (19)	42 (36)	<.001	527 (20)	110 (21)	>.99
Discharge, No. (%)									
SNF or LTCF	568 (23)	212 (28)	.10	746 (24)	34 (29)	>.99	629 (24)	151 (29)	.40
Home without assistance	518 (21)	91 (12)	<.001	592 (19)	17 (14)	>.99	533 (20)	76 (14)	.05

Abbreviations: AIS, Abbreviated Injury Severity Score; ED, emergency department; ICU, intensive care unit; LTCF, long-term care facility; NA, not applicable; NTDB, National Trauma Data Bank; RBC, red blood cell; SBP, systolic blood pressure; SNF, skilled nursing facility; SOFA, Sequential Organ Failure Assessment.

SI conversion factor: To convert base deficit to millimoles per liter of bicarbonate, multiply by 1.0.

^a^ Missing values of 5% or more are reported.

^b^ Wilcoxon rank sum test or Pearson χ^2^ test (all *P* values include Bonferroni corrections for multiple testing).

^c^ Body regions with frequency less than 10% are not shown and included face, upper extremity, and external injuries.

^d^ Defined as at least 2 body regions with serious injury (AIS ≥3) and at least 1 of the following physiological abnormalities during the first 2 calendar days of admission: Glasgow Coma Scale score of 8 or lower, base excess of 6 mEq/L or less of bicarbonate (to convert to millimoles per liter, multiply by 1.0), SBP less than 90 mm Hg, international normalized ratio of 1.4 or more, and aged 70 years or older.

^e^ Highest value documented during the first 48 hours of admission.

^f^ Highest score during the first 24 hours of admission.

^g^ Defined as being in the ICU with a SOFA score of 3 or higher on hospital days 13, 14, or 15.

In a post hoc comparison of concordant and discordant cases (eg, patients classified with sepsis by the automated clinical method but not by the NTDB vs patients classified by the NTDB but not the automated clinical method), patients classified by the automated clinical method had a greater number of ICU days and a greater number of days receiving mechanical ventilation and no meaningful differences in mortality or disposition compared with patients classified by the NTDB or the administrative method (eTable 2 and eTable 3 in Supplement 1). When the automated clinical and NTDB methods were in agreement, the automated clinical method identified sepsis several days earlier during the hospital course (eTable 4 in Supplement 1).

Chronic critical illness developed among 334 patients classified with sepsis using the automated clinical method (216 among those classified with sepsis on or before hospital day 10), 60 patients classified by the NTDB, and 209 by the administrative method (Table 4). The adjusted RR of chronic critical illness was more than 9-fold greater (9.9 [95% CI, 8.0-12.3]) among patients classified by the automated clinical method (5-fold greater [5.1 (95% CI, 4.1-6.3] when classified during the first 10 days), 5-fold greater (5.0 [95% CI, 3.4-7.3]) for the NTDB, and almost 5-fold greater (4.5 [95% CI, 3.6-5.6]) for the administrative method compared with patients without sepsis. The adjusted risk of in-hospital mortality was 30% higher (1.3 [95% CI, 1.0-1.6]) for the automated clinical method (60% higher for sepsis during the first 10 days), nearly 3-fold higher (2.7 [95% CI, 1.7-4.3]) for patients classified by the NTDB, and not significantly different (1.0 [95% CI, 0.7-1.2]) for patients classified by the administrative method compared with patients without sepsis.

**Table 4.** Associations of Sepsis With Chronic Critical Illness and In-Hospital Mortality According to 3 Sepsis Classification Methods

Outcome and sepsis classification method	No. (%)		RR (95% CI)	
	Sepsis with outcome	No sepsis with outcome	Unadjusted	Adjusted^a^
Chronic critical illness^b^				
Automated method	334 (45)	186 (8)	9.8 (8.0-12.1)	9.9 (8.0-12.3)
Automated method, onset of sepsis ≤10 d	216 (40)	304 (11)	5.1 (4.1-6.2)	5.1 (4.1-6.3)
NTDB	60 (51)	460 (15)	5.9 (4.0-8.6)	5.0 (3.4-7.3)
Administrative method	209 (40)	311 (12)	4.9 (4.0-6.1)	4.5 (3.6-5.6)
In-hospital mortality				
Automated method	174 (23)	463 (19)	1.3 (1.1-1.6)	1.3 (1.0-1.6)
Automated method, onset of sepsis ≤10 d	140 (26)	497 (19)	1.5 (1.2-1.9)	1.6 (1.3-2.1)
NTDB	42 (36)	595 (19)	2.3 (1.6-3.4)	2.7 (1.7-4.3)
Administrative method	110 (21)	527 (20)	1.1 (0.8-1.3)	1.0 (0.7-1.2)

Abbreviations: NTDB, National Trauma Data Bank; RR, relative risk.

^a^ Adjusted models included age in years, Charlson Comorbidity Index, Injury Severity Score, head maximum Abbreviated Injury Severity Score of 3 or more, multiple trauma, shock (base deficit ≥6 mEq/L of bicarbonate [to convert to millimoles per liter, multiply by 1.0] during first 24 hours or admission systolic blood pressure <90 mm Hg), admission Sequential Organ Failure Assessment score, and units of red blood cells transfused during the first 24 hours.

^b^ Chronic critical illness was defined as a Sequential Organ Failure Assessment Score of 3 or higher on hospital days 13, 14, or 15.

The automated clinical and NTDB methods were associated with a significantly lower hazard of recovery from organ dysfunction and greater hazard of in-hospital mortality among patients after sepsis diagnosis compared with patients at risk for sepsis according to each method (eFigure 3 and eTable 5 in Supplement 1).

## Discussion

In our study of more than 3100 adults with traumatic injury, we compared the epidemiology of posttraumatic sepsis according to 3 methods of classification: an automated method using clinical data from the EMR, the NTDB, and the administrative method as described by Angus et al.^12^ The first finding was that the classified prevalence of sepsis was markedly lower according to the NTDB (used in most trauma registries) compared with the automated clinical method and the administrative method. Second, all methods for sepsis classification appeared to have similar risk factors (with few exceptions) and associations with poor outcomes. However, agreement between classification methods was poor, which revealed that these methods do not reliably identify sepsis in the same patients. Third, we found that the automated clinical method, which identified the largest number of patients meeting Sepsis-3 criteria, had significant associations with chronic critical illness and in-hospital mortality. In contrast to the NTDB and administrative methods, the automated clinical method allowed for a more detailed description of Sepsis-3 criteria in the context of recovery from injury by identifying sepsis onset, the association between the decline of organ function and infection diagnosis, and sources of infection.

Our finding that the NTDB underestimated the prevalence of sepsis compared with the automated clinical method was not surprising and is likely due to its restrictive definition, requiring that sepsis be documented in the EMR. This is consistent with prior work from hospital-wide studies comparing explicit criteria with EMR-based methods.^14,16^ However, to date, this has not been shown in the trauma population, to our knowledge. The NTDB’s strong associations with in-hospital mortality can also be attributed to its use of explicit criteria. In a population-level study of more than 190 hospitals and more than 4 million records, claims data with explicit documentation of sepsis had the highest median estimated mortality of 25% compared with 10% using implicit administrative data (as used in the method by Angus et al^12^) and 16% using EMR-based clinical criteria.^16^ In the present study, the estimated RRs for sepsis-associated mortality followed a similar pattern. Another consideration is that prior versions of the NTDB definition for severe sepsis included explicit criteria for bacteremia.^10^ A recent study found that, among critically ill adults who met Sepsis-3 diagnostic criteria, those with bacteremia had proportionally higher mortality compared with those without bacteremia.^32^ This finding may partially explain the relatively lower mortality observed with the automated clinical method compared with the NTDB because the automated clinical method incorporates cultures from different body tissues. Although the NTDB and the automated clinical method had similarly strong associations with chronic critical illness, only a few patients at high risk were identified by the NTDB. That patients who were missed by the NTDB and captured by the automated clinical criteria had higher morbidity (more ICU days and more days receiving mechanical ventilation), with no differences in mortality, supports our claim that the NTDB is too restrictive. Finally, our findings suggest that an automated clinical approach may identify patients earlier in the disease process, which may be useful for sepsis detection research. These issues, combined with the fact that the NTDB uses administrative medical record review to ascertain documented sepsis, which may or may not be in line with Sepsis-3 and is a time-consuming and resource-intensive task, raise questions about the value of this approach.

The administrative method, which draws on explicit and implicit claims-based criteria, underestimated the severity of illness associated with posttraumatic sepsis because it was not associated with mortality. This finding likely reflects the low specificity of an implicit claims-based approach in the critically ill trauma population. The main issue is that the temporality of organ dysfunction (which may result from injury) relative to the documented infection (acquired during the hospital course) is not known, and the 2 claims may or may not be associated. In the trauma population, it is likely that the administrative method captures organ dysfunction associated with injury rather than, or in addition to, that from infection. The distribution of daily SOFA scores for the cohort supports this hypothesis. Among all patients, scores were highest during the first 3 to 4 days of hospitalization. However, SOFA scores among patients who met the automated clinical criteria for sepsis remained higher during the subacute phase of recovery (days 5-14). Not coincidentally, this was also the peak of sepsis onset. Those who met only administrative criteria for sepsis had lower scores by comparison during the same period, second only to patients who did not meet any method’s criteria for sepsis.

In addition to identifying more patients at high risk for poor outcomes after sepsis, the automated clinical method has other advantages compared with the NTDB and the administrative method. First, the temporal association between sepsis onset and chronic critical illness was only possible to delineate using EMR data. Although most sepsis events occur during the first 10 days of admission,^18^ infection and sepsis are associated with and likely cause many cases of chronic critical illness,^33^ while “sepsis recidivism” in the setting of chronic critical illness is also common.^4^ Thus, methods that lack information about sepsis onset may introduce bias from reverse causation when evaluating short-term outcomes such as chronic critical illness (this is less problematic for outcomes, such as death or disposition, which often terminate follow-up). The automated clinical method can also be used to identify understudied subgroups, such as patients with culture-negative sepsis, sources of infection, and the timing of organ dysfunction relative to antibiotic initiation—all opportunities for a more in-depth review of the circumstances and practices surrounding sepsis events among the critically injured population.

### Limitations

Our study has some limitations. It was performed at a single center, and results should be reproduced using data sets from other centers. Including less critically ill patients in this study would have resulted in a lower prevalence of sepsis. However, the burden of posttraumatic sepsis is highest among critically ill patients, and the use of more inclusive selection criteria would be unlikely to change the risk factors identified or the associations of sepsis with the major outcomes that we observed in this study. In addition, because there is likely an element of survival bias for infection and sepsis among critically ill patients, we cannot with certainty determine attributable mortality or the development of chronic critical illness directly to the sepsis event. Although the automated clinical method appears to be a superior option for sepsis classification compared with the NTDB or the administrative method, it has some limitations. Practices such as the timing and delivery of antibiotics may vary within and between institutions; this variation could affect whether patients meet all or only some criteria for Sepsis-3. Sepsis could also be misclassified using the automated clinical approach if worsening organ dysfunction associated with a noninfectious acute process prompted a diagnostic workup and empiric treatment. We recommend targeting culture-negative sepsis and subgroups meeting partial criteria for adjudication by medical record review as an efficient solution to concerns about misclassification. Although the automated clinical approach can be used to distinguish between culture-positive and culture-negative sepsis, identifying the true sources of infection in culture-negative sepsis likely requires in-depth medical record review.

Efficiently and accurately identifying life-threatening organ dysfunction from infection in the critically ill trauma population remains a challenge and reinforces the need for more longitudinal studies combining clinical parameters with diagnostic biomarkers. Future work would benefit from a shared sepsis data infrastructure, as recently described,^8^ as we continue to refine our methods for studying this disease process.

## Conclusions

The findings of this cohort study suggest that an automated classification method using clinical data from the EMR provides an efficient and robust approach to identifying sepsis in critically ill trauma patients. Using administrative data to identify sepsis for quality improvement analyses and retrospective research in the trauma population likely misclassifies a large proportion of patients at high risk with sepsis and should be replaced with methods that align with the Sepsis-3 definition, consider specific criteria for the context of injury, and provide information about sepsis onset, all of which are necessary to increase the yield of health services and population-based studies in this field.

## Notes

Supplement 1. **eFigure 1.** Flow Diagram of Study Inclusion and Exclusion Criteria **eTable 1.** Criteria Used to Classify Sepsis According to Three Methods **eFigure 2.** Distribution of Daily Sequential Organ Failure Assessment (SOFA) Scores Among Injured Adults **eTable 2.** Comparison of Outcomes Among Concordant and Discordant Sepsis Classifications: An Automated Clinical Method vs Angus Method **eTable 3.** Comparison of Outcomes Among Concordant and Discordant Sepsis Classifications: An Automated Clinical Method vs National Trauma Data Bank Method **eFigure 3.** Time to Recovery From Organ Dysfunction and Time to Death While In-hospital **eTable 4.** Characteristics and Outcomes Among Injured Adults Meeting Sepsis Criteria by Three Classification Methods, Stratified by Agreement Between Methods **eTable 5.** Time to Resolution of Organ Dysfunction and Time to In-hospital Death According to Two Sepsis Classification Methods
eMethods.

eReferences.

Supplement 2.
Data Sharing Statement
