# Supplementary material for: Defining Posttraumatic Sepsis for Population-Level Research
Source: JAMA Netw Open. 2023 Jan 18;6(1):e2251445. doi: 10.1001/jamanetworkopen.2022.51445 (PMC9857630; doi:10.1001/jamanetworkopen.2022.51445)
Supplement: Supplement 1. — eFigure 1. Flow Diagram of Study Inclusion and Exclusion Criteria eTable 1. Criteria Used to Classify Sepsis According to Three Methods eFigure 2. Distribution of Daily Sequential Organ Failure Assessment (SOFA) Scores Among Injured Adults eTable 2. Comparison of Outcomes Among Concordant and Discordant Sepsis Classifications: An Automated Clinical Method vs Angus Method eTable 3. Comparison of Outcomes Among Concordant and Discordant Sepsis Classifications: An Automated Clinical Method vs National Trauma Data Bank Method eFigure 3. Time to Recovery From Organ Dysfunction and Time to Death While In-hospital eTable 4. Characteristics and Outcomes Among Injured Adults Meeting Sepsis Criteria by Three Classification Methods, Stratified by Agreement Between Methods eTable 5. Time to Resolution of Organ Dysfunction and Time to In-hospital Death According to Two Sepsis Classification Methods eMethods. eReferences. [file jamanetwopen-e2251445-s001.pdf]

Supplementary Online Content

Stern K, Qiu Q, Weykamp M, O'Keefe G, Brakenridge SC. Defining posttraumatic sepsis for population-level research. *JAMA Netw Open.* 2023;6(1):e2251445.

doi:10.1001/jamanetworkopen.2022.51445

eFigure 1. Flow Diagram of Study Inclusion and Exclusion Criteria
eTable 1. Criteria Used to Classify Sepsis According to Three Methods
eFigure 2. Distribution of Daily Sequential Organ Failure Assessment (SOFA) Scores Among Injured Adults
eTable 2. Comparison of Outcomes Among Concordant and Discordant Sepsis Classifications: An Automated Clinical Method vs Angus Method
eTable 3. Comparison of Outcomes Among Concordant and Discordant Sepsis Classifications: An Automated Clinical Method vs National Trauma Data Bank Method
eFigure 3. Time to Recovery From Organ Dysfunction and Time to Death While In-hospital
eTable 4. Characteristics and Outcomes Among Injured Adults Meeting Sepsis Criteria by Three Classification Methods, Stratified by Agreement Between Methods
eTable 5. Time to Resolution of Organ Dysfunction and Time to In-hospital Death According to Two Sepsis Classification Methods
eMethods.
eReferences.

This supplementary material has been provided by the authors to give readers additional information about their work.

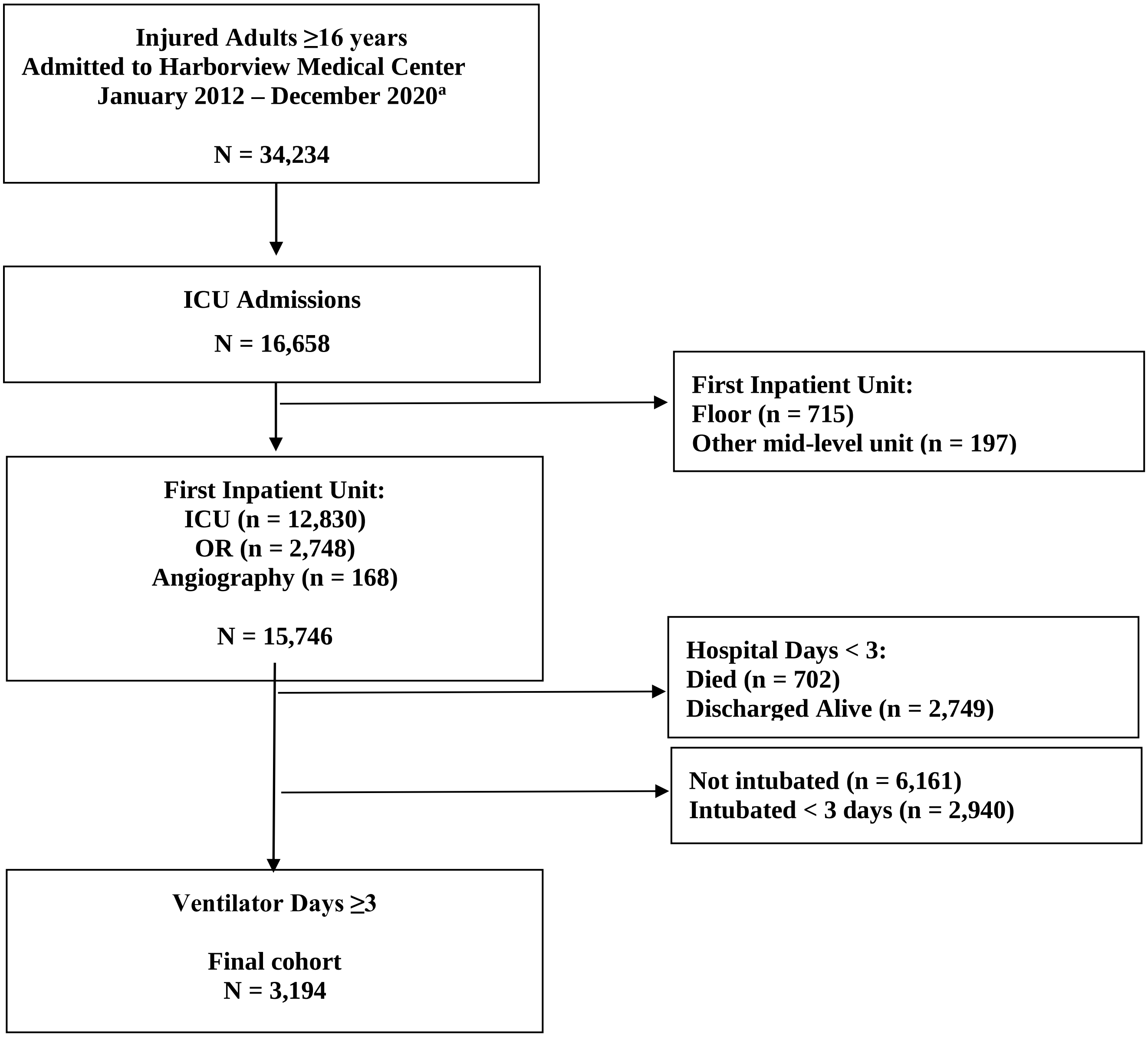

eFigure 1. Flow Diagram of Study Inclusion and Exclusion Criteria
[a]Study period does not include May through December of 2015.

© 2023 Stern K et al. *JAMA Network Open.*

eTable 1. Criteria Used to Classify Sepsis According to Three Methods

<table>
<tr><th>Automated Method</th><th>National Trauma Data Bank</th><th>Administrative Method</th></tr>
<tr>
<td>1. Body tissue culture ordered within 48 hours of an order for a new IV antibiotic[a]

AND

2. Any qualifying antibiotic sustained for at least 4 consecutive days[b]

AND

3. An increase in the modified daily SOFA score[c] of at least 2 points within 3 days prior to the qualifying culture and 3 days after</td>
<td>1. An obvious source of infection with bacteremia

AND

2. Two or more of the following:

• Temperature >38º C or <36º C
• WBC count >12,000/mm^3, or > 20% immature leukocytes
• Hypotension
• Evidence of hypoperfusion
• Anion gap or lactic acidosis or oliguria, or altered mental status</td>
<td>Implicit Criteria:
1. ICD code for infection

AND

2. ICD code for organ dysfunction

OR

Explicit Criteria:
ICD code for severe sepsis or severe sepsis with shock</td>
</tr>
</table>

[a]Body tissue included samples from CSF, respiratory, wound, tissue, fluid, abscess, genitourinary, and blood, and were not performed for surveillance. Antibiotics not considered to be therapeutic for sepsis after trauma did not meet IV antibiotic criteria: cefazolin, erythromycin, and gentamycin. Oral antibiotics that counted towards IV antibiotic criteria (even if not given IV): vancomycin and linezolid

[b]Includes cases when death or discharge occurred before the 4th consecutive antibiotic day

[c]Modified daily sequential organ failure assessment score (SOFA) excludes the neurologic component

Abbreviations: white blood cell (WBC), International Classification of Disease (ICD)

Multiple episodes of sepsis were collected using the “Automated” method; the first qualifying episode was used for the analysis in this study. Dates of sepsis diagnosis and whether there were multiple episodes was not available through the trauma registry, which relies on the NTDB method to assign sepsis as an outcome for the admission, nor was this information available through the Administrative method.

© 2023 Stern K et al. *JAMA Network Open.*

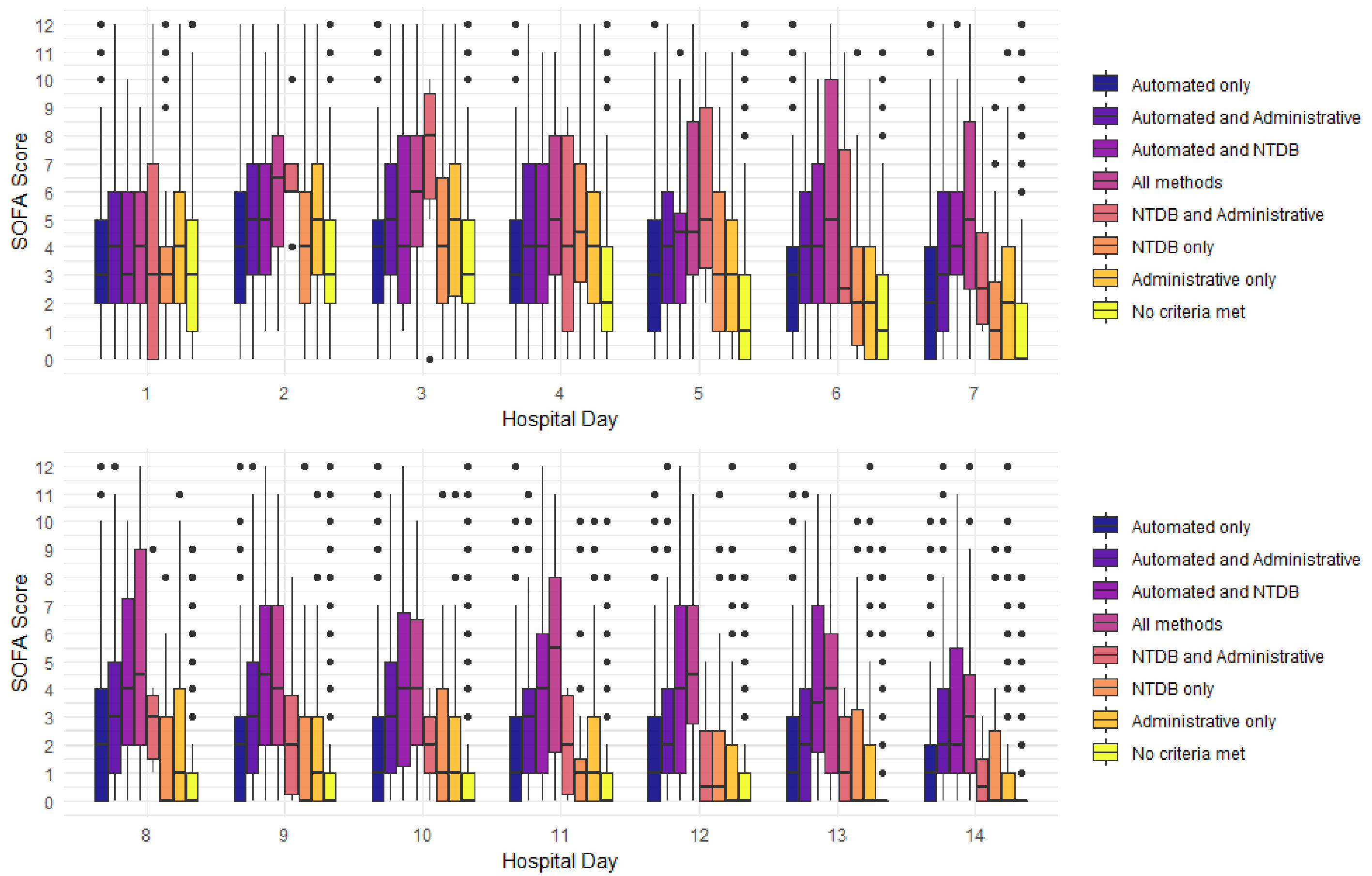

eFigure 2. Distribution of Daily Sequential Organ Failure Assessment (SOFA) Scores Among Injured Adults
Legend: Boxplots are stratified by sepsis classification according to three methods: an automated clinical method using data from the electronic medical record ("Automated"), an Administrative method ("Administrative"), and the National Trauma Data Bank ("NTDB").

© 2023 Stern K et al. *JAMA Network Open.*

eTable 2. Comparison of Outcomes Among Concordant and Discordant Sepsis Classifications: An Automated Clinical Method vs Angus Method

Outcome	Neither Criteria, N = 2,184	Angus Method Missed by Automated Method, N = 263	Automated Method Missed by Angus Method, N = 481	Both Criteria, N = 266	*P*[a]	
Ventilator Days	5 (3, 7)	9 (5, 16)	12 (8, 19)	18 (11, 31)	<0.001	
ICU Days	8 (5, 12)	14 (8, 21)	17 (12, 24)	25 (17, 39)	<0.001	
Chronic Critical Illness	133 (6.1%)	53 (20%)	178 (37%)	156 (59%)	<0.001	
Died	416 (19%)	47 (18%)	111 (23%)	63 (24%)	0.4	
Discharged to SNF/LTCH	492 (23%)	76 (29%)	137 (28%)	75 (28%)	0.025	
Discharged to Home without Assistance	476 (22%)	42 (16%)	57 (12%)	34 (13%)	<0.001	

[a]P-values from Kruskal-Wallis rank sum test, Pearson's Chi-squared test, or Fisher's exact test include Bonferroni adjustments for multiple testing
Abbreviations: intensive care unit (ICU), National Trauma Data Bank (NTDB), skilled nursing facility (SNF), long term care hospital (LTCH)

© 2023 Stern K et al. *JAMA Network Open.*

eTable 3. Comparison of Outcomes Among Concordant and Discordant Sepsis Classifications: An Automated Clinical Method vs National Trauma Data Bank Method

Outcome	Neither Criteria, N = 2,408	NTDB Missed by Automated Method, N = 39	Automated Method Missed by NTDB, N = 668	Both Criteria, N = 79	*P*[a]	
Ventilator Days	5 (3, 8)	6 (5, 11)	14 (8, 21)	16 (10, 26)	<0.001	
ICU Days	8 (6, 13)	14 (10, 21)	19 (13, 28)	24 (16, 36)	<0.001	
Chronic Critical Illness	175 (7.3%)	11 (28%)	285 (43%)	49 (62%)	<0.001	
Died	454 (19%)	9 (23%)	141 (21%)	33 (42%)	<0.001	
Discharged to SNF/LTCH	556 (23%)	12 (31%)	190 (28%)	22 (28%)	0.2	
Discharged to Home without Assistance	510 (21%)	8 (21%)	82 (12%)	9 (11%)	<0.001	

[a]P-values from Kruskal-Wallis rank sum test, Pearson's Chi-squared test, or Fisher's exact test include Bonferroni adjustments for multiple testing
Abbreviations: intensive care unit (ICU), skilled nursing facility (SNF), long term care hospital (LTCH)

© 2023 Stern K et al. *JAMA Network Open.*

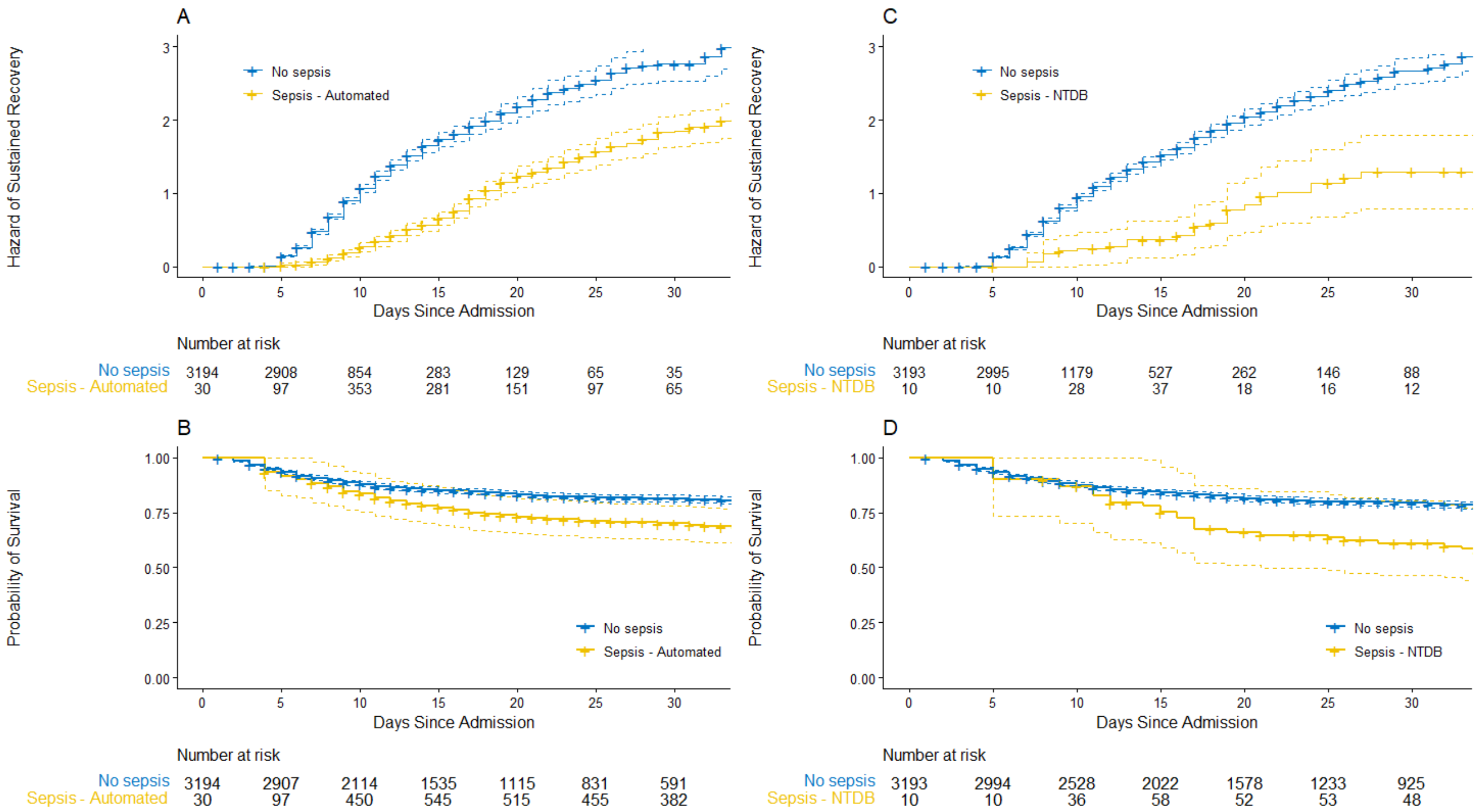

eFigure 3. Time to Recovery From Organ Dysfunction and Time to Death While In-hospital. Panels A and B depict hazard curves for recovery (sustained daily SOFA score <3 for 3 or more consecutive days or until discharge) and survival curves for patients classified with or without sepsis by an Automated method. Panels C and D depict the recovery and survival curves for patients classified with or without sepsis by the National Trauma Databank method. Estimates account for sepsis as a time-varying event. Deaths were censored as no recovery in the time to resolution of organ dysfunction analysis.

© 2023 Stern K et al. *JAMA Network Open.*

eTable 4. Characteristics and Outcomes Among Injured Adults Meeting Sepsis Criteria by Three Classification Methods, Stratified by Agreement Between Methods

	All methods, N = 38	Automated Only, N = 440	NTDB Only, N = 33	Administrative Only, N = 257	Two Methods Overlap[a], N = 275	No sepsis, N = 2,151
**Pre-Hospital**						
Age, n (IQR)	54 (32, 66)	48 (33, 62)	55 (36, 71)	53 (33, 63)	52 (33, 65)	49 (30, 64)
Male, n (%)	25 (66)	348 (79)	23 (70)	198 (77)	212 (77)	1,574 (73)
Comorbidity Index ≥3[b], n (%)	8 (21)	77 (18)	10 (30)	61 (24)	67 (24)	478 (22)
**Injury**						
Blunt-Force Mechanism, n (%)	29 (76)	408 (93)	27 (82)	233 (91)	252 (92)	1,877 (87)
Injury Severity Score, n (IQR)	37 (29, 43)	30 (25, 42)	28 (22, 36)	29 (25, 43)	29 (24, 43)	27 (20, 36)
Body Region AIS≥3[b], n (%)						
Head	10 (26)	254 (58)	11 (33)	116 (45)	107 (39)	1,053 (49)
Chest	20 (53)	246 (56)	15 (45)	149 (58)	165 (60)	983 (46)
Abdomen	21 (55)	109 (25)	11 (33)	75 (29)	81 (29)	403 (19)
Spine	8 (21)	91 (21)	5 (15)	65 (25)	63 (23)	424 (20)
Lower Extremity	21 (55)	121 (28)	13 (39)	96 (37)	102 (37)	622 (29)
Polytrauma[c], n (%)	31 (82)	280 (64)	21 (64)	169 (66)	174 (63)	1,193 (55)
**Physiology & Interventions**						
Base Déficit[e], (mmol/L), n (IQR)	7 (5, 12)	5 (3, 8)	6 (4, 7)	6 (4, 10)	7 (4, 10)	5 (3, 8)
ED SBP <90mmHg, n (%)	8 (22)	57 (13)	8 (25)	53 (21)	43 (16)	239 (11)
RBC Transfusion, n (%)	25 (66)	180 (41)	15 (45)	114 (44)	140 (51)	792 (37)
RBC Units First 24 hours, n (IQR)	9 (2, 18)	3 (1, 6)	2 (1, 4)	4 (2, 8)	4 (2, 8)	2 (1, 5)
APACHE II[f], n (IQR)	32 (28, 34)	27 (22, 32)	28 (24, 32)	29 (24, 33)	29 (24, 34)	27 (22, 31)
Emergency Laparotomy, n (%)	21 (55)	95 (22)	12 (36)	64 (25)	70 (25)	322 (15)

© 2023 Stern K et al. *JAMA Network Open.*

	All methods, N = 38	Automated Only, N = 440	NTDB Only, N = 33	Administrative Only, N = 257	Two Methods Overlap[a], N = 275	No sepsis, N = 2,151
**Outcomes**						
**Sepsis Onset - Automated**	**8 (6, 11)**	**7 (6, 10)**	**-**	**-**	**7 (6, 10)**	**-**
**Sepsis Onset - NTDB**	**11 (8, 14)**	**-**	**10 (5, 13)**	**-**	**11 (9, 18)**	**-**
ICU Days, n (IQR)	30 (20, 41)	17 (12, 24)	14 (10, 21)	14 (8, 21)	23 (16, 36)	8 (5, 12)
Ventilator Days, n (IQR)	18 (13, 32)	12 (7, 19)	6 (5, 10)	9 (5, 16)	17 (10, 28)	5 (3, 7)
Chronic Critical Illness[g], n (%)	27 (71)	156 (35)	9 (27)	51 (20)	153 (56)	124 (6)
Died, n (%)	13 (34)	91 (21)	8 (24)	46 (18)	71 (26)	408 (19)
SNF or LTCF, n (%)	12 (32)	127 (29)	10 (30)	74 (29)	75 (27)	482 (22)
Home w/o assistance, n (%)	4 (11)	52 (12)	6 (18)	40 (16)	37 (13)	470 (22)

Continuous data are presented as the median (interquartile range); discrete data as number (%); missing values ≥5% are reported
[a]Met sepsis criteria by two different methods (e.g., NTDB and Automated or NTDB and Administrative or Automated and Administrative). The values reported for hospital day of sepsis onset are limited to patients who specifically overlapped between the Automated and NTDB criteria. The date for sepsis onset according to the NTDB isthe complication date documented in the trauma registry.
[b]Charlson comorbidity index
[c]Body regions with frequency <10% are not shown and included face, upper extremity, and external injuries
[d]Polytrauma was defined as at least 2 body regions with serious (AIS≥3) injury and at least 1 of the following physiologic abnormalities (GCS≤8, base excess ≤6, systolic blood pressure <90mmHg, INR ≥1.4, Age ≥70) during the first 2 calendar days of admission
[e]Highest value documented during the first 48 hours of admission
[f]Highest score during the first 24 hours of admission
[g]Defined as being in the ICU with a SOFA score of 3 or higher on hospital days 13, 14, or 15.
Abbreviations: abbreviated injury severity score (AIS), emergency department (ED), intensive care unit (ICU), long term care facility (LTCF), National Trauma Data Bank (NTDB), Red blood cell (RBC), Skilled nursing facility (SNF), systolic blood pressure (SBP)
SI conversion factors: To convert mmHG to Pascals, multiply by (133.3).

© 2023 Stern K et al. *JAMA Network Open.*

eTable 5. Time to Resolution of Organ Dysfunction and Time to In-hospital Death According to Two Sepsis Classification Methods

	Recovery from Organ Dysfunction		In-Hospital Mortality	
Method	Unadjusted HR (95% CI)	Adjusted HR (95% CI)	Unadjusted HR (95% CI)	Adjusted HR (95% CI)
Automated	0.6 (0.5, 0.6)	0.5 (0.4, 0.5)	2.0 (1.6, 2.4)	2.0 (1.6, 2.5)
NTDB	0.5 (0.4, 0.7)	0.5 (0.4, 0.8)	2.6 (1.7, 4.0)	2.8 (1.8, 4.3)

Cox Proportional Hazards models adjusted for age, sex, head AIS greater than 3, ISS, polytrauma, RBC units transfused during the first 24 hours, and admission SOFA scores.
A "hazard" of recovery >1 indicates relatively faster recovery whereas values <1 indicate slower or prolonged recovery times.

© 2023 Stern K et al. *JAMA Network Open.*

eMethods.

Sepsis Definitions:

We defined sepsis consistent with the Sepsis-3 consensus guidelines as a clinically suspected infection associated with acute worsening of organ dysfunction.[1] To identify post-traumatic sepsis retrospectively, we used the CDC's adult sepsis surveillance criteria[2] with *a priori* modifications using readily obtainable EMR data to address specific classification challenges specific to the trauma population. We required that all of the following be present: 1) an order for a new IV or qualifying oral antibiotic, not administered within the previous 48 hours and excluding antibiotics used for surgical prophylaxis, 2) a body tissue culture was ordered within 48 hours of antibiotic initiation, 3) a qualifying antibiotic was sustained for at least 4 consecutive days, or until death or discharge, and 4) a 2-point increase in the maximum daily sequential organ failure assessment (SOFA) score occurred within 3 days before and 3 days after the qualifying culture. We restricted our criteria to hospital acquired infections, which we defined as cultures obtained on or after the third hospital day.[3] We used a modified version of the SOFA score that omits the Glasgow Comma Scale (GCS) because traumatic brain injury and the administration of sedating medications are common confounders in critically ill trauma patients.[4,5] If laboratory values or clinical data required for the calculation of a specific SOFA score component (e.g., bilirubin) were missing, we assumed organ function was normal (i.e., missing not at random) and assigned a point value of 0. We also used a broader window to capture deteriorating organ function than described in precedent guidelines because organ dysfunction is already prevalent in the severely injured and the added contribution from an infectious process may be subtle or develop more gradually in this setting.[6,7] For descriptive purposes, we further classified sepsis identified by the automated method as "culture-proven" or "culture-negative." Cultures that met the CDC's National Healthcare Safety Network (NHSN) criteria for hospital acquired infections[8] based on bacterial growth were classified as culture-proven; those that did not meet NHSN criteria were classified as culture-negative.

Identifying Infection Source, and Classifying Culture-positive and Culture-negative Sepsis:

We developed a text-sorting algorithm to parse results from body tissue cultures and screen those meeting the CDC's National Healthcare Safety Network's (NHSN) criteria for hospital acquired infections.[8] Culture data including the source, sampling method used to obtain tissue samples, and culture results were extracted from the electronic medical record into an Excel file, and read into R statistical software. In R, we used text sorting and filtering tools to create columns containing quantitative (colony forming units or CFUs), semi-quantitative (1+ through 4+), and qualitative (organism name) information on bacterial or fungal growth. The NHSN criteria includes specifications on the type and quantity of bacterial growth that qualifies as an infection for different body tissues (e.g., blood, urine, respiratory) and different thresholds for quantitative data meeting infection criteria based on the sampling method. We classified the following sources of infection: cerebral spinal fluid, respiratory, genitourinary, blood, and surgical or traumatic wound (included cultures labeled as tissue, fluid, intraabdominal, abscess, and wound). Cultures meeting NHSN criteria for infection that also met antibiotic and organ dysfunction criteria were classified as "culture positive sepsis." Cultures not meeting NHSN criteria but meeting antibiotic and SOFA criteria were classified as "culture negative sepsis."

Application of the Administrative Method:

We ascertained sepsis using two additional classification methods: the NTDB and the methodology described by Angus and colleagues (i.e., "Administrative").[9] The NTDB classification of severe sepsis is performed by a non-clinician administrator and includes manual chart review for documentation of sepsis and laboratory evidence of organ dysfunction or hypoperfusion.[10] The Administrative method includes explicit and implicit criteria for sepsis based on international classification of disease (ICD) codes.[9] ICD codes corresponding to severe sepsis or septic shock met explicit criteria for sepsis. To identify sepsis using implicit criteria, we applied the original ICD-9 codes for infection and organ dysfunction described by Angus et al[9] to all records with ICD-9 codes, which applied to admission records from 2012 through 2015. We then identified ICD-10 codes corresponding to the original ICD-9 codes using a crosswalk file, or general equivalence mapping, provided by the Centers for Medicare and Medicaid Services.[11] Using the cross-walked set of ICD-9 and ICD-10 codes, we applied the same Administrative criteria to records from 2016 through 2020. We reviewed all codes manually to ensure complete capture and overlap between relevant ICD-9 and ICD-10 diagnoses.

© 2023 Stern K et al. *JAMA Network Open.*

eReferences.

1. Singer M, Deutschman CS, Seymour CW, et al. The Third International Consensus Definitions for Sepsis and Septic Shock (Sepsis-3). *JAMA*. 2016;315(8):801-810. doi:10.1001/jama.2016.0287

2. Rhee C, Klompas M. Sepsis trends: increasing incidence and decreasing mortality, or changing denominator? *J Thorac Dis*. 2020;12(Suppl 1):S89-S100. doi:10.21037/jtd.2019.12.51

3. Vincent JL. Nosocomial infections in adult intensive-care units. *The Lancet*. 2003;361(9374):2068-2077. doi:10.1016/S0140-6736(03)13644-6

4. Eriksson J, Eriksson M, Brattström O, et al. Comparison of the sepsis-2 and sepsis-3 definitions in severely injured trauma patients. *Journal of Critical Care*. 2019;54:125-129. doi:10.1016/j.jcrc.2019.08.019

5. Aperstein Y, Cohen L, Bendavid I, et al. Improved ICU mortality prediction based on SOFA scores and gastrointestinal parameters. *PLoS One*. 2019;14(9). doi:10.1371/journal.pone.0222599

6. Eguia E, Cobb AN, Baker MS, et al. Risk factors for infection and evaluation of Sepsis-3 in patients with trauma. *Am J Surg*. 2019;218(5):851-857. doi:10.1016/j.amjsurg.2019.03.005

7. Minei JP, Cuschieri J, Sperry J, et al. The changing pattern and implications of multiple organ failure after blunt injury with hemorrhagic shock. *Crit Care Med*. 2012;40(4):1129-1135. doi:10.1097/CCM.0b013e3182376e9f

8. NHSN | CDC. Published December 15, 2021. Accessed April 5, 2022. http://www.cdc.gov/nhsn/index.html

9. Angus DC, Linde-Zwirble WT, Lidicker J, Clermont G, Carcillo J, Pinsky MR. Epidemiology of severe sepsis in the United States: analysis of incidence, outcome, and associated costs of care. *Crit Care Med*. 2001;29(7):1303-1310. doi:10.1097/00003246-200107000-00002

10. NTDS - Dataset Dictionary. Accessed April 7, 2022. http://155.100.188.85/dataElements/datasetDictionary.html

11. CMS' ICD-9-CM to and from ICD-10-CM and ICD-10-PCS Crosswalk or General Equivalence Mappings - MyNBER. Accessed April 14, 2022. https://data.nber.org/data/icd9-icd-10-cm-and-pcs-crosswalk-general-equivalence-mapping.html

© 2023 Stern K et al. *JAMA Network Open.*
