# Supplementary material for: Defining Posttraumatic Sepsis for Population-Level Research
Source: JAMA Netw Open. 2023 Jan 18;6(1):e2251445. doi: 10.1001/jamanetworkopen.2022.51445 (PMC9857630; doi:10.1001/jamanetworkopen.2022.51445)
Supplement: Supplement 2. — Data Sharing Statement [file jamanetwopen-e2251445-s002.pdf]

Data Sharing Statement

Stern. Defining Posttraumatic Sepsis for Population-Level Research. *JAMA Netw Open*. Published January 18, 2023. doi:10.1001/jamanetworkopen.2022.51445

Data
Data available: Yes
Data types: Deidentified participant data, Data dictionary
How to access data: Requests for analyzable deidentified participant data can be sent to (sbrakenr@uw.edu).
When available: With publication

Supporting Documents
Document types: Statistical/analytic code
How to access documents: Requests for statistical/analytic code can be sent to (sbrakenr@uw.edu).
When available: With publication

Additional Information
Who can access the data: Data and analytic code will be made available to researchers whose proposed use of the data has been approved.
Types of analyses: A deidentified, analytic dataset will be made available for replication or secondary analyses.
Mechanisms of data availability: The data will be made available after approval of a proposal.
